# DGPT news: Fritz Külz Award 2024

**DOI:** 10.1007/s00210-025-04126-7

**Published:** 2025-04-17

**Authors:** Edda S. F. Matthees

**Affiliations:** Institute of Molecular Cell Biology, Center for Molecular Biomedicine, University Hospital Jena, Hans-Knöll-Str.2, 07745 Jena, Germany

**Keywords:** G protein-coupled receptors, Signaling regulation, Biased agonism

G protein-coupled receptors (GPCRs) are prominent drug targets, due to their high accessibility at the plasma membrane and broad involvement in physiological processes (Hauser et al. 2017). As this family of receptors lack intrinsic enzymatic activity, GPCRs signal through interaction with downstream effectors, primarily heterotrimeric G proteins composed of a Gα subunit and a Gβγ dimer. The downstream signaling is regulated by a small family of kinases, namely GPCR kinases (GRKs), which phosphorylate intracellular receptor domains, facilitating β-arrestin recruitment. These scaffolding proteins mediate different functions, including receptor desensitization and internalization (Gurevich and Gurevich 2019).

In previous years, G protein signaling and β-arrestin-supported functions have often been considered to be independent. The observation of reduced adverse opioid effects in β-arrestin2 knockout mice (Bohn et al. 1999) spurred research into biased agonism, aiming to selectively activate either G protein- or β-arrestin-mediated GPCR pathways to minimize off-target effects by developing biased agonists (Gesty-Palmer et al. 2006; DeWire and Violin 2011).

β-arrestin-supported functions critically dependent on receptor phosphorylation, highlighting the importance of GRKs, which were previously understudied due to the limited availability of tools. Notably, however, in vitro studies revealed that GRK2/3 interact with the membrane-associated Gβγ subunits (Pitcher et al. 1995) to facilitate their translocation to the plasma membrane, where the activated receptors reside. More recently, we and others revealed differential GRK dependence among GPCRs for their regulation, utilizing advanced knockout systems to study individual GRK isoforms without endogenous background (Drube et al. 2022; Haider et al. 2022; Kawakami et al. 2022). While some receptors exhibit specificity for the cytosolic GRK2/3 subfamily, others rely on the membrane-tethered GRK5/6, while some show no isoform preference among the ubiquitously expressed GRKs.

**Fig. 1:**
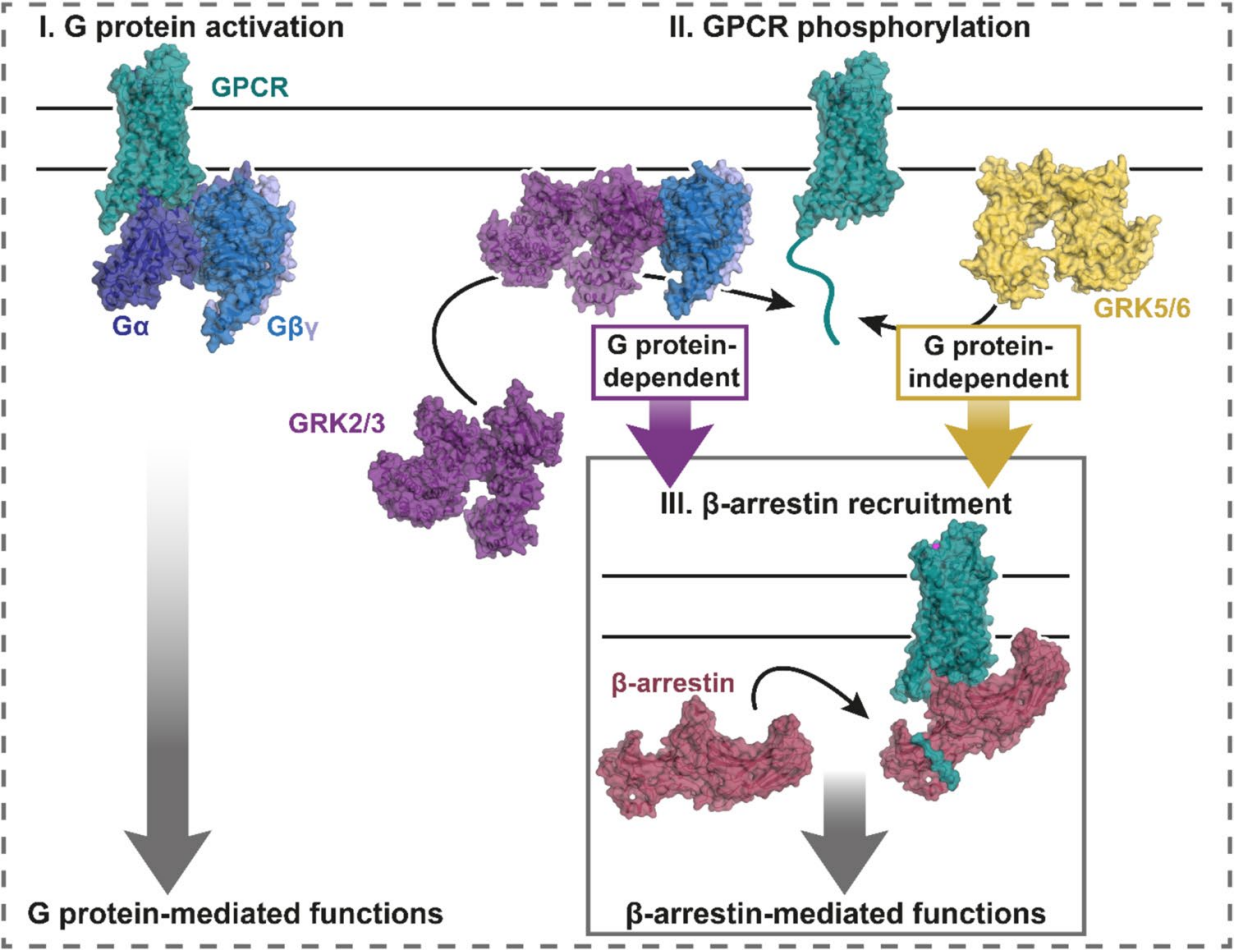
G protein- and β-arrestin-mediated functions are only independent of each other in the context of GRK5/6-, but not GRK2/3-mediated phosphorylation. GPCR stimulation typically initiates G protein activation (I), leading to the dissociation of Gα and Gβγ subunits, which mediate distinct downstream effects. As part of the signaling regulation, GPCR phosphorylation (II) promotes β-arrestin recruitment (III), facilitating functions such as receptor desensitization and internalization. The cytosolic GRK2/3 subfamily, however, requires free Gβγ subunits to translocate to the plasma membrane where the active receptor resides, while GRK5/6 are already membrane-localized. Hence, GRK2/3-mediated phosphorylation is dependent on G protein activation. This lays an important mechanistic framework for the development of biased agonists of GPCRs. Understanding the GRK specificity of a receptor is crucial, as it determines the potential for selectively modulating GPCR signaling pathways. Utilized PDB files for the visualization: 60S9, 3CIK, 8JPB, 6UP7, 6PJX

Distinct GRK recruitment mechanisms between the GRK subfamilies and the GRK specificity of GPCR regulation led us to investigate this interplay between G protein-mediated signaling and β-arrestin-supported functions (Matthees et al. 2024). Using GRK knockout cells in combination with GRK mutants, incapable of G protein interaction, we demonstrated that GRK2/3–Gβγ interaction is essential for β-arrestin recruitment to several receptors. Further, inhibiting G protein activation indeed significantly impaired both β-arrestin recruitment and receptor internalization as demonstrated for a GRK2/3-specific GPCR (Matthees et al. 2024). Hence, while GRK5/6-mediated receptor phosphorylation is G protein-independent, GRK2/3-mediated receptor functions rely on the interaction with free Gβγ dimers (Fig. 1).

Understanding this GRK specificity is crucial for developing β-arrestin-biased agonists. For GRK5/6-dependent receptors, G protein activation is inconsequential as these GRKs are constitutively membrane-localized. However, for GRK2/3-dependent receptors, kinase localization is a key consideration for selectively activating β-arrestin-supported pathways. When receptors are regulated by all four ubiquitously expressed GRKs, developing β-arrestin-biased agonists is mechanistically possible. However, one needs to investigate whether the difference in available GRKs to mediate receptor phosphorylation—due to their distinct recruitment mechanisms—leads to different β-arrestin-supported downstream functions.

With this work, we provide a mechanistic framework to determine the potential for selectively modulating GPCR signaling pathways by considering the GRK dependence on liberated Gβγ. Our findings highlight the importance of considering receptor– “GRK bias” when designing strategies to selectively induce G protein- or β-arrestin-mediated functions.
